# Underpinning Starch Biology with *in vitro* Studies on Carbohydrate-Active Enzymes and Biosynthetic Glycomaterials

**DOI:** 10.3389/fbioe.2015.00136

**Published:** 2015-09-07

**Authors:** Ellis C. O’Neill, Robert A. Field

**Affiliations:** ^1^Department of Plant Sciences, University of Oxford, Oxford, UK; ^2^Department of Biological Chemistry, John Innes Centre, Norwich Research Park, Norwich, UK

**Keywords:** starch, glucans, phosphorylase, self-assembly, nanotechnology, synthetic biology

## Abstract

Starch makes up more than half of the calories in the human diet and is also a valuable bulk commodity that is used across the food, brewing and distilling, medicines and renewable materials sectors. Despite its importance, our understanding of how plants make starch, and what controls the deposition of this insoluble, polymeric, liquid crystalline material, remains rather limited. Advances are hampered by the challenges inherent in analyzing enzymes that operate across the solid–liquid interface. Glyconanotechnology, in the form of glucan-coated sensor chips and metal nanoparticles, present novel opportunities to address this problem. Herein, we review recent developments aimed at the bottom-up generation and self-assembly of starch-like materials, in order to better understand which enzymes are required for starch granule biogenesis and metabolism.

## Introduction

Starch makes the highest calorific contribution to the human diet (U.S. Department of Agriculture, 2012) and is a bulk commodity, with a global market of billions of tons per year (Ellis et al., 1998). We cannot meet the ever-increasing demand for starch-based products solely by further commitment of agricultural land to conventional starch-based crops – as well as increases in crop yield, new ways to produce modified starch-based materials are also needed (Diouf, 2009). In order to achieve this goal, the production of modified starches *in planta* is an attractive alternative to post harvest modification (Jobling, 2004) – i.e., moving the diversification of starch functionality from the chemical plant into the crop plant. To this end, better understanding of the enzymes involved in starch metabolism and those used in industrial starch modification is needed.

## Starch Structure and Metabolism

**Figure 1:**
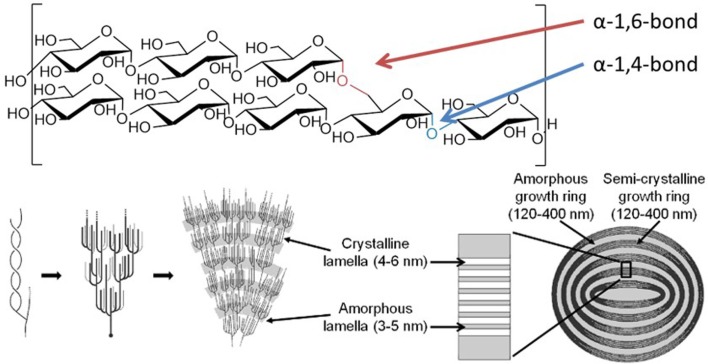
**Structure of the starch granule**. Starch granules are composed of α-1,4-glucans with some α-1,6-branches, placing them parallel to each other, allowing double helix formation. The branches are specifically placed, giving regions of branching, known as amorphous lamella, and regions of exclusively linear chains which form crystalline lamella, and these lamella form defined growth rings. Specific enzymes are needed to synthesize and degrade this highly ordered insoluble structure. Coultate (2002) – Reproduced by permission of The Royal Society of Chemistry.

Starch granules comprise linear α-1,4-glucans with periodic α-1,6-branches, giving long chains capable of wrapping around each other to form double helical arrangements, which stack side by side, forming alternating layers of highly ordered liquid crystalline lamella interspersed with amorphous regions (Figure 1) (Waigh et al., 1998). This self-organizing nanostructure makes the surface of a starch granule highly resistant to enzymatic attack, requiring specialized enzymes to initiate degradation.

In order to engineer precisely defined starch structures, we need to understand the metabolic enzymes involved, insights into which are provided by continued research in the model plant *Arabidopsis thaliana* (Santelia and Zeeman, 2011). Multiple enzymes are required to create the correct morphology of the starch granule, and the specific role of individual enzyme isoforms is not well-understood (Ball and Morell, 2003; Tetlow, 2011). For example, the correct *synthesis* of granular starch counter-intuitively requires two *degradative* isoamylases (Bustos et al., 2004). Storage starch is formed in the amyloplasts of specialist storage organs, such as the grain or tuber, while transitory starch is stored in the leaf chloroplasts, as a carbon source for photosynthetic cells at night. The two plastids use different suites of enzymes to attack the starch granule (Smith et al., 2005; Smirnova et al., 2015). In the storage organs, the granule is enzymatically hydrolyzed (Radchuk et al., 2009), ultimately releasing glucose; in photosynthetic organs, the transitory starch granule is initially phosphorylated by specific glucan kinases, before being broken down into short maltooligosaccharides (Fettke et al., 2009). There are several isoforms of each class of enzyme involved in starch synthesis and breakdown, but differences in their action are generally interpreted in terms of the expression of the gene and protein in question, rather than of the enzyme activity *per se*. The complication of enzymatic reactions taking place on an insoluble starch substrate accounts for these crude approximations, a situation that needs to be addressed in order to inform plant engineering/synthetic biology studies.

## Current Status on the Enzymatic Degradation of Insoluble Starch

The enzymology of the starch granule actually takes place at a solid–liquid interface, rather than solely in the solution phase used in most experiments. This can have profound effects on the reaction; for example, the reaction rate of granule-bound starch synthase is enhanced on crystalline amylopectin (Edwards et al., 1999). There are few enzyme studies involving crystalline maltodextrins (Hejazi et al., 2009) or purified starch granules (Edner et al., 2007), but even these studies measured discreet product formation, not breakdown of the starch granule. Electron microscopy clearly shows that the initial attack on the surface alters the granule by disrupting the insoluble packing of the glucan chains (Sun and Henson, 1990), undermining endpoint kinetic assessments. Direct monitoring of purified starch granules during degradation, either using electron microscopy (Planchot et al., 1995) or synchrotron radiation (Tawil et al., 2011), provides snapshots of these processes during the degradation of the granule. Clearly such experiments provide information on structures, but not on reaction rate as such.

## *In Vivo* Modification of Starch

The source of starch can have a marked impact on physico-chemical properties that are important for industrial uses. For instance, cereal starch is virtually free of phosphate, while potato starch has high phosphate content, with increased viscosity and decreased crystallization, factors important in the paper industry (Blennow et al., 2003). For commercial applications, starches may need to be chemically modified – by fragmentation, oxidation, or esterification – although this is costly and potentially hazardous. Production of fit-for-purpose modified starches *in planta* is therefore an attractive alternative (Jobling, 2004) and has been achieved in a number of ways. For instance, the gelatinization properties of the starch can be controlled by using genetic engineering to alter the ratio of linear amylose to branched amylopectin (Visser et al., 1991; Jobling et al., 2002). Removal of the glucan water dikinase, which adds phosphate to starch chains during degradation, from potato tubers produced a starch that was resistant to degradation during storage (Lorberth et al., 1998). By replacing the plant disproportionating enzyme, which transfers glucans between chains during starch degradation, with its bacterial homolog the complex soluble heteroglycan could be essentially bypassed during transitory starch degradation (Ruzanski et al., 2013). It has also been possible to engineer a vaccine-displaying starch by fusing starch-binding proteins to known antigens in algal chloroplasts (Dauvillée et al., 2010). However, it is not easy to predict outcomes at the whole plant level due to poor knowledge of individual enzyme functions (Stanley et al., 2011), coupled with compensatory mechanisms and genetic redundancy or partially overlapping enzymatic capabilities.

## *In Vitro* Synthesis and Modification of Starches

A number of approaches have been investigated to produce natural and non-natural starch-like materials *in vitro*. To simulate the branch point of a starch granule, specific glucans could be linked synthetically using “click chemistry,” producing known length chains with known positioning of the branch point (Marmuse et al., 2005; Nepogodiev et al., 2007). Longer unbranched amylose polymers can be synthesized enzymatically in solution, either by the debranching of amylopectin with isoamylase (Harada et al., 1972) or by the extension of acceptor glucans with amylosucrase or glucan phosphorylase (Yanase et al., 2007). Amylosucrase has been used to synthesize dendritic nanoparticles, based on a glycogen core (Putaux et al., 2006); in combination with branching enzyme, a highly branched glucan can be accessed from sucrose (Grimaud et al., 2013). Amylomaltases, which transfer glucans from one chain onto another, can also be used to generate fluorinated glucans (Tantanarat et al., 2012) and to modify starches in order to make thermoreversible gels (Kaper et al., 2005). Interestingly, phosphorylases also display some promiscuity toward the donor substrate and they have been used to prepare a range of modified glucans (O’Neill and Field, 2015). 2-Deoxy-maltooligosaccharides (Klein et al., 1982) have been synthesized from d-glucal in the presence of Pi, which can then be phosphorolyzed to synthesize 2-deoxy-α-glucose- 1-phosphate. Deoxy- and fluoro-glucose moieties can also be transferred onto glycogen by glucan phosphorylase, but in low yield (Withers, 1990). Alternative sugar-1-phosphates can be utilized by glucan phosphorylase, including those derived from xylose (Nawaji et al., 2008b), mannose (Evers and Thiem, 1997), glucosamine (Nawaji et al., 2008a), *N*-formyl-glucosamine (Kawazoe et al., 2010), and glucuronic acid (Umegatani et al., 2012), although the products were all isolated after a single residue extension, indicating that these sugars cannot be bound in the acceptor site of the enzyme for further extension. Under certain conditions, and with careful choice of phosphorylase, further extension has been achieved to make a polyglucosamine, analogous to the α-steroisomer of chitosan (Kadokawa et al., 2015). By addition of both glucosamine and glucuronic acid onto a glycogen core, pH-responsive amphoteric hydrogels could be synthesized (Takata et al., 2015).

Plant phosphorylases, with their lack of allosteric regulation (Fukui et al., 1982), have proven more useful than the mammalian equivalent in the synthesis of long chain amylose derivatives, for instance, by extension of glucan immobilized on chitosan (Kaneko et al., 2007) or on polystyrene (Loos and Müller, 2002), or by twining polysaccharides around a hydrophobic core to assemble a macromolecular complex, such as amylose-wrapped lipid (Gelders et al., 2005).

## Engineering Insoluble Starch Surfaces for Enzymatic Analysis

When amylose is synthesized in solution, it self-assembles into crystalline structures (Buleon et al., 2007). Using electron microscopy, this type of crystalline material can be seen after extension of glycogen particles using amylosucrase (Putaux et al., 2006) and X-ray diffraction shows that it has assembled into head-to-tail, B-type starch (Potocki-Veronese et al., 2005). Many physiologically important enzymatic reactions occur on surfaces and their analysis is far from trivial. A range of techniques have been deployed to address this point, including mass spectrometry, radioactivity, and fluorescence-based assays (Gray et al., 2013). Quartz crystal microbalance technology has also been used to monitor real-time extension of amylopectin by phosphorylase (Murakawa et al., 2007) and to provide associated kinetic information (Nishino et al., 2004). The recent development of plant oligosaccharide microarrays also enables high throughput analysis of carbohydrate-active enzymes (Pedersen et al., 2012).

While it has been possible to detect glycosylation reactions on surfaces using surface plasmon resonance (SPR), this technique has typically been deployed to assess non-catalytic lectin binding to sugars (Karamanska et al., 2008). Direct measurement of *trans*-glycosylation reactions on SPR sensors has been achieved (Cle et al., 2008, 2010), but the enzymes to which this technique has been applied use soluble substrates and act in solution. Any kinetic analysis has to therefore take into account the unnatural nature of the surface-immobilized substrates. Starch-active enzymes would benefit markedly from studies using immobilized substrates, which mimic the insoluble surface upon which they naturally act.

The plant phosphorylase, PHS2, can rapidly synthesize α-1,4-glucans in solution and, by immobilizing their acceptor oligosaccharide substrates, insoluble glucan surfaces could be developed (O’Neill et al., 2014). The SPR surfaces behaved in a manner dependent on the density of the immobilized glucan. When relatively dilute, the glucan surface behaved in the same way as in solution; when a denser surface was used, the material produced took on the pattern of enzyme resistance seen in natural granular starch. These results indicate that the amyloglucan polymer created by PHS2 on the high density surface formed a macromolecular architecture, which may be crystalline, rendering the surface resistant to enzymatic digestion. These surfaces could be used to assay proteins that interact with the starch granule to ascertain binding kinetics in classical SPR experiments. Starch-degrading enzymes are normally assayed in solution, which is not a good analog of their natural insoluble granule substrate. Classic enzyme kinetics cannot be utilized to study reactions on an insoluble surface and care will be needed to differentiate between the binding of soluble enzymes to the insoluble surface or the reaction itself as the rate limiting step. This new surface technology offers the prospect of a more informative assay to provide kinetic information on starch surface degradation.

**Figure 2:**
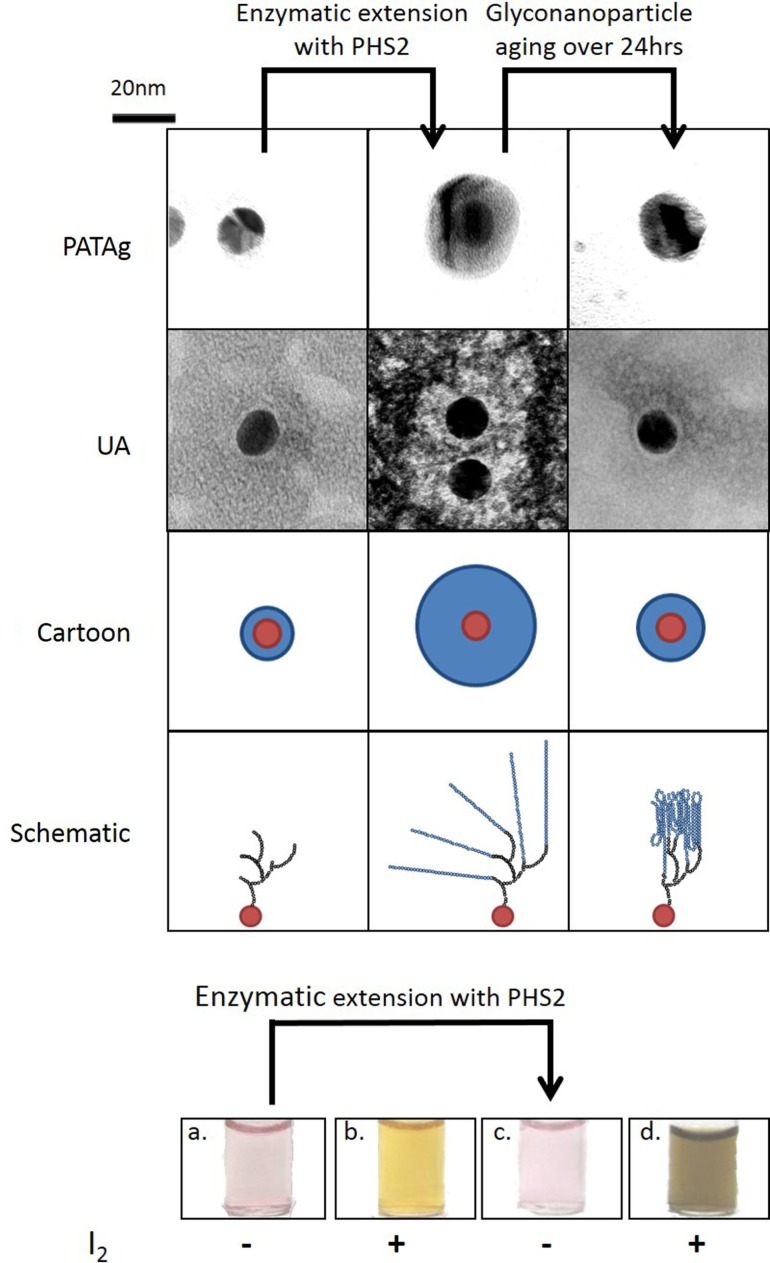
**Synthesis of 3D glucan structures on the surface of gold nanoparticles**. Gold nanoparticles with an immobilized glucan on the surface were imaged with TEM after PHS2-mediated extension with two staining methods (PATAg and UA). As illustrated in the cartoon, the carbohydrate (blue) on the nanoparticles (red), increases after treatment with PHS2 and rearranges to form a thinner layer after 24 h. The schematic shows that the starting glucan (black) is extended by AtPHS2 (blue) and then rearranges by forming intra and inter-chain interactions to produce a more condensed overall structure. Before extension the gold nanoparticles (a) stained weakly with iodine (b), but after PHS2-mediated extension (c) they stained strongly (d), indicating the formation of ordered starch helices. Adapted from O’Neill et al. (2014).

While a quantifiable 2D system is relevant for kinetic analysis of starch-active enzymes, a third dimension is required to represent the true spatial arrangement of the starch granule. The uniform shape and size and defined physical and chemical properties of gold nanoparticles make them useful materials for the study of biological interactions (Saha et al., 2012). Gold nanoparticles can be simultaneously used to display carbohydrates and to provide visual output of interactions (Marin et al., 2015), using the same photophysical effects exploited in conventional SPR studies, to give a change in color from red to purple. Glucan primers immobilized on gold nanoparticles could be extended substantially by PHS2 (O’Neill et al., 2014). The resulting nanoparticle-based glucan surfaces were subject to aging, with the glucan layer appearing to reorganize and assemble into a much more tightly packed structure over the course of ~12 h (Figure 2). This type of glyconanoparticle has potential for the analysis of enzymes that naturally act on starch granules, and may serve as models for bottom-up, *in vitro* synthetic biological approaches to biocompatible inorganic surfaces.

## Conclusion

Reflecting the global need for sustainability and a drive toward environmentally benign manufacturing practices, new ways to produce bulk commodities, such as starch, are key. In order to achieve this goal, better information about how nature handles (bio)chemistry across the liquid–solid interface is necessary. Here, we have highlighted the potential of bottom-up synthetic biology approaches to produce starch-like surfaces in a format suitable for both structural analysis and real-time kinetic assessment of enzyme action thereon. These glyconanotechnology methods will help to pave the way to a more complete understanding of natural starch metabolism and may be used to inform understanding and exploitation of the natural processes, including the recapitulation of starch production in non-starch producing organisms.

## Conflict of Interest Statement

The authors declare that the research was conducted in the absence of any commercial or financial relationships that could be construed as a potential conflict of interest.
